# Predictive impact of human papillomavirus circulating tumor DNA in treatment response monitoring of HPV‐associated cancers; a meta‐analysis on recurrent event endpoints

**DOI:** 10.1002/cam4.6377

**Published:** 2023-07-26

**Authors:** Abbas Karimi, Tohid Jafari‐Koshki, Mojtaba Zehtabi, Farzaneh Kargar, Tarik Gheit

**Affiliations:** ^1^ Department of Molecular Medicine, Faculty of Advanced Medical Sciences Tabriz University of Medical Sciences Tabriz Iran; ^2^ Department of Statistics and Epidemiology, Faculty of Health Tabriz University of Medical Sciences Tabriz Iran; ^3^ Hematology and Oncology Research Center Tabriz University of Medical Sciences Tabriz Iran; ^4^ Department of Medical Biotechnology, Faculty of Medical School Tabriz University of Medical Sciences Tabriz Iran; ^5^ Epigenomics and Mechanisms Branch, International Agency for Research on Cancer (IARC) Lyon France

**Keywords:** circulating tumor DNA, HPV ctDNA, HPV‐related cancer, human papillomavirus, survival analysis

## Abstract

**Background:**

HPV infection can cause cancer, and standard treatments often result in recurrence. The extent to which liquid biopsy using HPV circulating tumor DNA (HPV ctDNA) can be used as a promising marker for predicting recurrence in HPV‐related cancers remains to be validated. Here we conducted a systematic review and meta‐analysis to assess its effectiveness in predicting treatment response.

**Methods:**

We conducted a systematic literature search of online databases, including PubMed, Embase, Scopus, and the Cochrane Library, up to December 2022. The goal was to identify survival studies that evaluated the potential of plasma HPV ctDNA at baseline and end‐of‐treatment (EoT) in predicting recurrence of related cancers. Hazard ratios were estimated directly from models or extracted from Kaplan–Meier plots.

**Results:**

The pooled effect of HPV ctDNA presence on disease recurrence was estimated to be HR = 7.97 (95% CI: [3.74, 17.01]). Subgroup analysis showed that the risk of recurrence was HR = 2.17 (95% CI: [1.07, 4.41]) for baseline‐positive cases and HR = 13.21 (95% CI: [6.62, 26.36]) for EoT‐positive cases. Significant associations were also observed between recurrence of oropharyngeal squamous cell carcinoma (HR = 12.25 (95% CI: [2.62, 57.36])) and cervical cancer (HR = 4.60 (95% CI: [2.08, 10.17])) in plasma HPV ctDNA‐positive patients.

**Conclusions:**

The study found that HPV ctDNA detection can predict the rate of relapse or recurrence after treatment, with post‐treatment measurement being more effective than baseline assessment. HPV ctDNA could be used as a surrogate or incorporated with other methods for detecting residual disease.

## Graphical abstract

The study showed that detecting HPV ctDNA can predict the rate of relapse or recurrence after treatment, with post‐treatment measurement appearing to be superior to baseline assessment in recurrence prediction. This highlights the potential of HPV ctDNA as a surrogate or in combination with other methods for detecting residual disease.

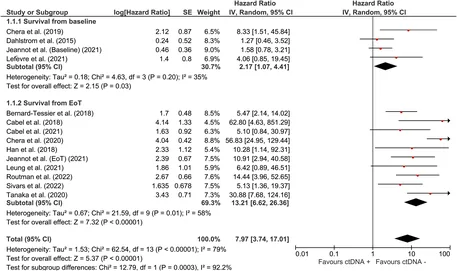

## 1 INTRODUCTION

Among viruses that contribute to malignant transformation, the human papillomavirus (HPV) has attracted major attention. This virus is the culprit for about one‐third of infection‐related cancers and plays remarkable roles in different steps of cancer evolution.^1^ Infections with mucosal HPV types can contribute to carcinomas originating from anogenital mucosa, and head and neck, mostly from the oropharynx.^2^, ^3^, ^4^ These diverse types of cancer burdens attributed to HPV are potentially preventable by screening and vaccination programs.^5^ Based on agents classified by the IARC Monographs, 14 high‐risk HPV types (HPV 16, 18, 31, 33, 35, 39, 45, 51, 52, 56, 58, 59, 66, and 68) are considered Groups 1 and 2A carcinogen.^6^ Nearly 75% of all HPV‐associated squamous cell carcinomas and more than 90% of all adenocarcinomas are attributable to high‐risk subtypes 16, 18, 31, 33, and 45.^1^, ^7^ HPV16 is known to be the most common type of HPV that is associated with oropharyngeal cancer and alone is responsible for more than 90% of HPV‐positive oropharyngeal cancers, which is significantly higher than its prevalence in cervical cancer.^8^

Nowadays in the setting of major HPV‐related cancers like cervical cancer (CC), head and neck squamous cell carcinoma (HNSCC), and anal squamous cell carcinoma (ASCC) the preferred standard‐of‐care therapeutic approach includes the combination of platinum‐based chemotherapy and radiation.^9^, ^10^, ^11^ Although chemoradiotherapy (CRT) alone has shown benefits for many years, this method faces some considerable challenges like resistance and adverse events making reliable response prediction biomarkers to be identified essentially.^10^, ^11^ A significant proportion of patients with advanced HNSCC, cervical, vulval, and anal cancers undergoing CRT have a persistent or recurrent disease^12^; which can be monitored by biomarkers obtained from blood‐based liquid biopsies.^13^, ^14^ This non‐invasive cost‐effective biopsy method can provide dynamic information on tumor development by giving biological data of heterogeneous tumor tissue and the metastatic tissues simultaneously.^15^

HPV‐related tumor cells usually contain several copies of the HPV genome, as a single genome and multiple tandem head‐to‐tail repeats in humans, as well as in episomal form.^16^ Small fragments of the double‐stranded HPV genome, as reported for genomic ctDNA, are released into the bloodstream by apoptosis, necrosis, pyroptosis, phagocytosis, cytoplasmic swelling (oncosis), or ferroptosis.^17^ These fragments, referred to as HPV circulating tumor DNA (HPV ctDNA), are detected using ddPCR for HPV E7/E6 sequences. The detection of these fragments have higher specificity as a predictive biomarker for relapse compared to the detection of HPV integration sites.^18^ Recent meta‐analyses have reported the diagnostic accuracy of HPV ctDNA in HPV‐associated cancers.^19^, ^20^, ^21^, ^22^ Gu et al. suggested that the detection of HPV ctDNA in patients with CC appears to be a highly specific but relatively sensitive diagnostic parameter.^21^ Jensen et al. indicated that plasma HPV ctDNA is a promising tool for surveillance in patients with HPV‐related HNSCC.^22^ Campo et al. concluded cell‐free human papillomavirus‐DNA (cfHPV‐DNA) represents a potential confirmatory and complementary assay for the positive results of positron emission tomography/computed tomography (PET/CT) scan.^20^ In addition, Balachandra et al. and Galati et al. indicated that HPV16 E6 antibodies and circulating HPV‐16 DNA are valuable candidate blood‐based biomarkers of HPV‐associated cancers.^19^, ^23^ Both next‐generation sequencing (NGS) and digital droplet polymerase chain reaction (ddPCR) as high throughput techniques are major methods to study HPV ctDNA and have been demonstrated to be more accurate than quantitative PCR (qPCR).^13^ The use of ddPCR, or a bead‐based assay such like the E7‐MPG, in liquid biopsy improves the diagnostic value of HPV ctDNA significantly.^21^, ^23^, ^24^ There is a discrepancy in the data published on HPV ctDNA by OMIC techniques as a predictive biomarker in the surveillance of HPV‐related cancers.^25^, ^26^ This meta‐analysis aims to pool the recent publications to evaluate whether the detection of plasma HPV ctDNA can be used as a reliable tool for treatment response monitoring in HPV‐related cancers by considering the recurrence endpoints.

## 2 MATERIALS AND METHODS

### 2.1 Search strategy and inclusion criteria

**FIGURE 1:**
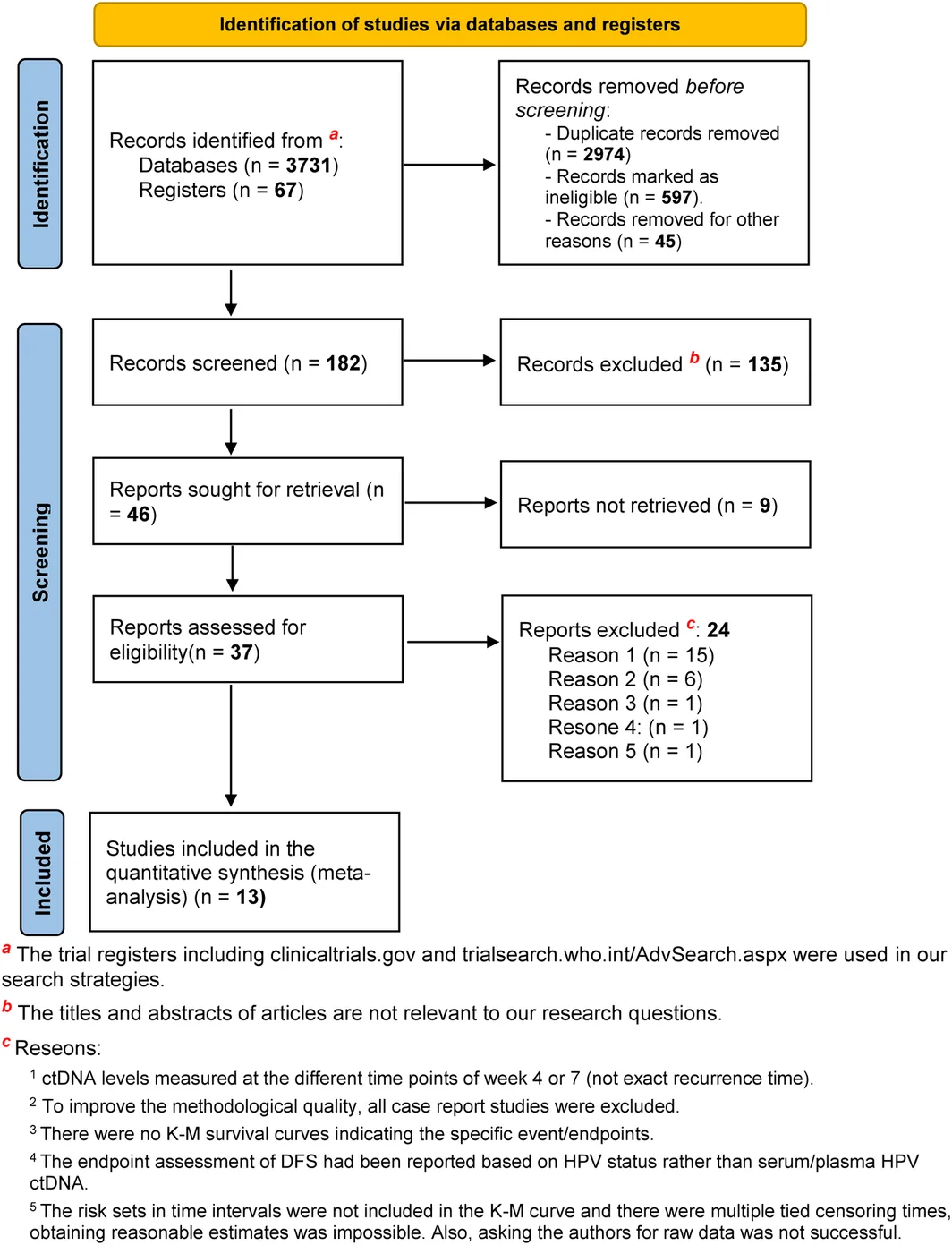
PRISMA flow diagram of the study. In this study, we used the “PRISMA 2020 flow diagram” template which included searches of only databases and registers.

Online databases of PubMed, Embase, Scopus, and the Cochrane Library were systematically searched to find all relevant studies on HPV ctDNA and CC, HNSCC, and ASCC up to December 2022. A combination of subsequent MeSH terms, including “Human Papillomavirus Viruses”, “Circulating Tumor DNA”, “Cell‐Free Nucleic Acids”, “Uterine Cervical Neoplasms”, “Anus Neoplasms”, “Head and Neck Neoplasms”, “Disease‐Free Survival”, “Progression‐Free Survival”, “Minimal Residual Disease”, “Survival Analysis” and “Kaplan–Meier Estimate” were used to search the literature. Moreover, search terms that had no available MeSH terms included “HPV ctDNA”, “Relapse‐Free Survival”, “Recurrence‐Free Survival”, “Locoregional Relapse‐Free Survival”, “Distant Metastasis‐Free Survival (DMFS)”, “Liquid Biopsy Analysis”, “Prognostic Value”, “Prognostic Factor”, “Prognostic Indicator”, “Prognostic and Predictive Biomarkers”, “Recurrence Risk”, “Predictive for Outcome”. The flow diagram of the meta‐analysis and exclusion criteria are shown in Figure 1. The flow diagram was prepared based on The Preferred Reporting Items for Systematic reviews and Meta‐Analyses (PRISMA) 2020 statement guideline.^27^

### 2.2 Data extraction

The following information was obtained from each study: first author, year of publication, cancer type, HPV ctDNA detection/quantitation methods, number of patients in the time‐to‐event analysis, treatment strategies, and recurrent event endpoints data. Chemotherapy with radiation therapy (CRT), chemotherapy only, and radiation therapy only were chosen among other standard treatment regimens. To assess the efficacy of treatment strategies on recurrent event endpoints, we assumed that the widely used endpoints for tumor assessments—progression‐free survival (PFS), disease‐free survival (DFS), recurrence‐free survival (RFS), and event‐free survival (EFS)—represent the same concept, based on studies documenting their interchangeability. The Food and Drug Administration (FDA) defines DFS and PFS as below for approval of cancer drugs and biologics; DFS is defined as the time from randomization or treatment until disease recurrence or death from any cause. PFS is defined as the time from randomization or treatment until objective tumor progression or death, whichever occurs first.^28^ DFS also known as relapse‐free survival (RFS) is the time from treatment to the relapse (local, regional, and distant).

Based on the assumptions of these documents, we conclude that DFS, PFS, RFS, and EFS are the time elapsed between the date of initiation or end of treatment and the date of cancer relapse or tumor recurrence. All endpoints are estimated by the Kaplan–Meier (K–M) plots and survival curves. Plasma/serum HPV ctDNA values of pre‐therapeutic (baseline), end‐of‐treatment (EoT), and recurrence at follow‐up were used for estimations of these endpoints across studies.

We use two types of analysis for time‐to‐event data: one for the positivity of HPV ctDNA at EoT and the second for HPV ctDNA value at baseline. To have homogeneous Kaplan–Meier (K–M) data, all reported values were converted to hazard ratio (HR). To minimize underestimation of HPV ctDNA detection, documents that used ddPCR and HPV sequencing (HPV‐seq) methods were chosen for final analysis.

Titles and abstracts of the papers were carefully reviewed, as articles with similar keywords but content unrelated to the purpose, articles without an appropriate treatment strategy, unclear HPV DNA detection methods, case report studies, and papers assessing only the primary efficacy endpoint of overall survival (OS), and paper having HPV ctDNA value just in recurrence time were excluded from the study.

### 2.3 Statistical analysis

To conduct a meta‐analysis on survival studies, we needed estimates on hazard ratios comparing the groups and their standard errors (SE). However, survival studies report different information and most of the time, required data for meta‐analysis are not directly reported. We retrieved data on HR and SE directly or by back‐calculation from confidence intervals (CI) for those studies that had reported this information. For those that had reported the K–M curve along with risk sets in groups at specific time intervals, we estimated the required information by using approximation methods available in the literature.^29^ Some studies neither report HR estimates directly nor represent the risk sets on K–M curves, but show censoring times on the K–M curve by symbols. For these studies, we used GetData Graph Digitizer software (getdata‐graph‐digitizer.com) to extract original data from the K–M curves and then used the Cox PH model to estimate HRs and corresponding SEs. As indicated by heterogeneity indices, a random‐effects model was used to calculate pooled HR and corresponding CI. Pooled estimates and related plots were obtained by Review Manager software (RevMan) version 5.4.^30^

## 3 RESULTS

### 3.1 Study selection

The initial search, according to the detailed list of the search terms yielded the detection of 3798 records (Figure 1). After the duplicates are identified, 597 publications were assessed for systematic search by filtering to further narrow down these results. Then, 183 records were screened in the title and abstract. Forty‐seven records remained eligible for full‐text assessment. After full‐text assessments, 13 studies meeting the search criteria were eligible for final review. An overview of the studies having eligible time‐to‐event analysis data and their main clinical characteristics are shown in Tables 1 and 2.

**TABLE 1:** Overview of the studies stating time‐to‐event analysis in HPV‐related cancer.

No.	Authors and Year	Country	Tumor type	HPV ctDNA quantification	Cases No. in time‐to‐event analysis	Surrogate endpoint assessment (pre‐post‐treatment marker)	Medical treatment for outcome measurement
1	Leung et al. (2021)^31^	Canada	LACC	HPV‐seq	16	**PFS**: EOT detectable HPV ctDNA	CRT
2	Jeannot et al. (2021)^18^	France	CC	ddPCR	40	**PFS**: EOT detectable HPV ctDNA	CRT, Neo‐chemotherapy
						**PFS**: Baseline detectable HPV ctDNA	
3	Cabel et al. (2021)^32^	France	LACC	ddPCR	14	**DFS**: EOT detectable HPV ctDNA	CRT
4	Lefèvre et al. (2021)^33^	Denmark	SCCA	ddPCR	45	**DFS**: Baseline high and low pHPV	CRT
5	Tanaka et al. (2020)^34^	Japan	HNSCC	ddPCR	35	**EFS**: EOT detectable HPV ctDNA 16	Radiotherapy with or without chemotherapy
6	Chera et al. (2020)^35^	USA	OPSCC	ddPCR	115	**RFS**: EOT detectable HPV ctDNA	CRT
7	Chera et al. (2019)^36^	USA	OPSCC	ddPCR	48	**RDFS** ^a^: Baseline favorable and unfavorable clearance HPV ctDNA16	CRT
8	Bernard‐Tessier et al. (2018)^37^	France	SCCA	ddPCR	36	**PFS**: EOT detectable HPV ctDNA	Chemotherapy
9	Cabel et al. (2018)^38^	France	ASCC [locally advanced]	ddPCR	18	**DFS:** EOT detectable HPV ctDNA	CRT
10	Dahlstrom et al. (2015)^26^	USA	OPSCC [OPC]	qPCR	99	**PFS**: Baseline detectable circulating HPV DNA	Chemotherapy and Radiotherapy^b^
11	Han et al. (2018)^39^	Canada	LACC	ddPCR	19	**PFS**: EOT detectable HPV ctDNA	CRT
12	Routman et al. (2022)^40^	USA	OPSCC	ddPCR	159	**RFS**: EOT detectable HPV ctDNA	**Adjuvant radiation therapy**
13	Sivars et al. (2022)^41^	Sweden	LACC	ddPCR	18	**PFS**: EOT detectable HPV ctDNA	CRT and EBRT

Abbreviations: CC: cervical cancer; CRT: chemoradiotherapy; DFS: disease‐free survival; EBRT: external beam radiation therapy; EFS, event‐free survival; HNSCC: squamous cell carcinoma of the head and neck; LACC: locally advanced cervical cancer; OPSCC: oropharyngeal squamous cell carcinoma; PFS: progression‐free survival; RDFS: regional disease‐free survival; RFS: recurrence‐free survival; SCCA: Squamous cell carcinoma of the anus; SCCA: squamous cell carcinomas of the anal canal [Anal squamous cell carcinoma (SCCA)].

^a^ RDFS: was measured from the first CRT dose and defined as the time to detect or develop persistent or recurrent disease in the cervical lymph nodes.

^b^ During and after treatment, patients underwent regular clinical and radiologic examinations with a team of specialists in head and neck cancer, including surgeons, radiation oncologists, and medical oncologists.

**TABLE 2:** Main outcomes of the included studies.

No	Study type	Study type	Sample	HPV ctDNA monitoring outcome
1	Leung et al.	Prospective clinical trial	Plasma	Detectable plasma HPV ctDNA at EOT timepoint was associated with shorter PFS
2	Jeannot et al.	Prospective cohort (the multicenter trial)	Serum	Significant association between HPV ctDNA at EOT and PFS was obtained
				Baseline HPV ctDNA levels was not associated with PFS
3	Cabel et al.	Prospective study	Serum/Plasma	HPV‐ctDNA detection at the end of CRT and/or during follow‐up was associated with shorter DFS
4	Lefèvre et al.	Prospective study	Plasma	High versus low median pretreatments plasma HPV levels had different DFS
5	Tanaka et al.	^a^Not specified	Plasma	No detectable ctHPV16DNA was associated with longer EFS
6	Chera et al.	Prospective biomarker clinical trial	Plasma	Actuarial 2‐year RFS rates between undetectable HPV ctDNA at all surveillance time points vs patients with at least one abnormal HPV ctDNA level were 30% for patients with an abnormal HPV ctDNA valus during post‐treatment surveillance and 100% for patients with negative HPV ctDNA value
7	Chera et al.	Prospective biomarker study	Plasma	Favorable clearance of ctHPV16DNA was associated with 100% RDFS
8	Bernard‐Tessier et al.	Multicenter prospective single‐arm trial	Serum	Detectable HPV ctDNA at the end of chemotherapy was associated with poorer PFS
9	Cabel et al.	Prospective study	Plasma/Serum	HPV ctDNA detection after CRT was strongly associated with shorter DFS
10	Dahlstrom et al.	Prospective cohort study	Plasma/serum	Negative and positive HPV ctDNA levels at the pretreatment timepoint were not significantly associated with longer PFS
11	Han et al.	Prospective multicenter study	Plasma	Patients with undetectable HPV ctDNA at CRT completion time had significantly longer PFS than those with detectable levels.
12	Routman et al.	Prospective study or part of clinical trials	Serum	Detectable HPV ctDNA level after surgical treatment was significantly associated with shorter RFS
13	Leung et al.	Not specified	Plasma	Patients with detectable HPV ctDNA at EOT timepoint had significantly shorter PFS compared to those with negative HPV ctDNA values

^a^ The patients were newly diagnosed with HPV16‐related HNSCC and underwent radiotherapy with or without chemotherapy.

### 3.2 Variability in study design and quality assessment

Of 13 studies included in the meta‐analysis, 5 studies were related to cervical, 3 to anal, and 6 to head and neck cancers. Both squamous cell carcinoma (SCCA) and anal squamous cell carcinoma (ASCC) are considered neoplasms of the anal canal. In the case of HNSCC, most of the records were HPV‐positive oropharyngeal squamous cell carcinoma (OPSCC). Only one study indicated HPV16‐related HNSCC patients treated by radiotherapy with or without chemotherapy.

In the included studies, different surrogate endpoint assessments including PFS, DFS, EFS, RFS, and RDFS (Regional Disease‐Free Survival) indicated the same idea and concept for treatment response assessment, thanks to the same meaning, all of them were considered as an endpoint assessment in the final analysis. Of 13 studies, 11 studies used EoT [at end of treatment/post‐treatment], 3 studies used baseline, and one study reported both EoT and baseline presence/absence HPV ctDNA for estimating disease relapse/recurrence prediction. Two studies used HPV ctDNA 16 levels in response assessment and outcome. Since the HPV16 ctDNA biomarker is highly specific and sensitive to the detection HPV positive cancer; we considered these studies in the final analysis too. Twelve studies used high‐throughput assays of ddPCR and HPV sequencing for HPV ctDNA quantitation. As shown in Table 1, among 13 studies, nine studies used EoT detectable HPV ctDNA as the predictor of recurrence, two studies used baseline detectable HPV ctDNA, one study used both EoT and baseline detectable HPV ctDNA, one study used baseline favorable and unfavorable clearance HPV ctDNA. Quality assessment of the studies based on the QUADAS‐2 indicated that the variations mentioned above meet our selection criteria and the overall risk of bias was low. The 13 included studies fulfilled the criterion for the items of patient selection, medical treatment for outcome measurement, endpoint assessment, HPV ctDNA quantification, and tumor.

### 3.3 HPV ctDNA meta‐analysis

**FIGURE 2:**
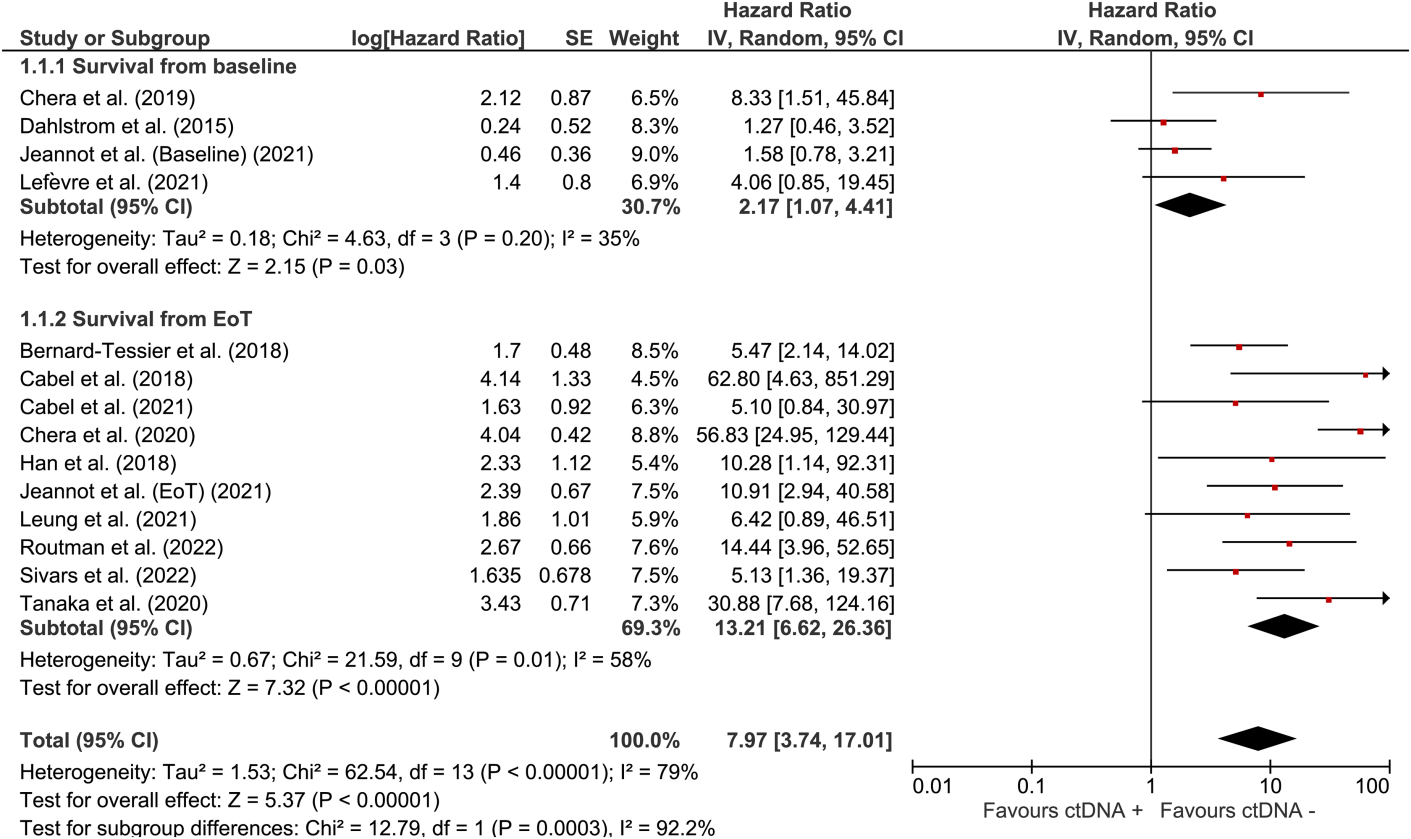
Risk of subsequent HPV‐related cancer recurrence for HPV ctDNA positive cases at baseline and EoT timepoints.

**FIGURE 3:**
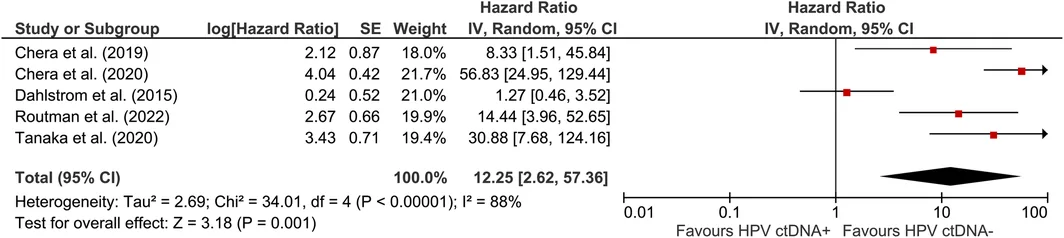
Risk of subsequent OPSCC recurrence in HPV ctDNA positive cases.

**FIGURE 4:**
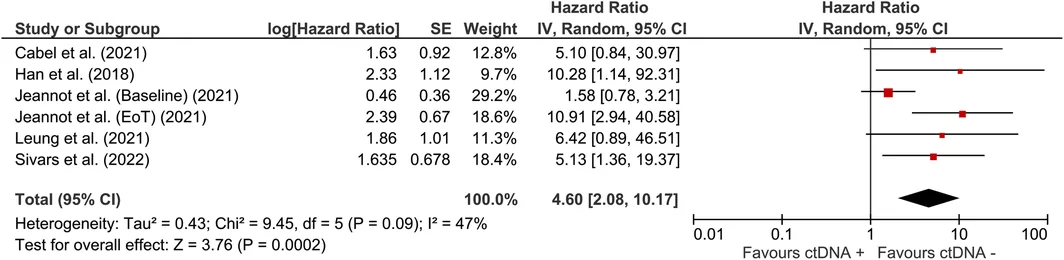
The risk of subsequent cervical cancer for HPV ctDNA positive cases.

A total of 662 patients were included according to time‐to‐event analysis data from 13 HPV‐related studies. The pooled estimate of the effect of HPV ctDNA presence on disease recurrence in HPV‐related cancers was HR = 7.97 (95% CI: [3.74, 17.01]) (Figure 2). Subgroup analysis was carried out to find the source of heterogeneity. As indicated by reduced heterogeneity in subgroups, the estimates from baseline and EoT studies were basically different. The estimated risk of cancer recurrence for HPV ctDNA positivity at baseline and EoT timepoints were HR = 2.17 (95% CI: [1.07, 4.41]) and HR = 13.21 (95% CI: [6.62, 26.36]), respectively (Figure 2). The pooled estimate for OPSCC (Figure 3) was HR = 12.25 (95% CI: [2.62, 57.36]) for HPV ctDNA positive cases. As shown in Figure 4, the pooled effect for CC was estimated as HR = 4.60 (95% CI: [2.08, 10.170]) for HPV ctDNA+ cases.

### 3.4 Sensitivity analysis results

For the case that all studies included in the analysis (Figure 2), after removing Chera et al. (2020), *I* ^2^ was reduced from 79%, 35%, and 58% to 62%, 35%, and 0% for total, baseline, and EoT studies, respectively. Removal of the other studies showed negligible effect on heterogeneity (<2% reduction in *I* ^2^). For OPSCC incidence (Figure 3), after the removal of Dahstrom et al. (2015), *I* ^2^ dropped from 88% to 47%. None of these studies had an appreciable impact on the pooled estimate of the studies with respect to direction or significance.

## 4 DISCUSSION

HPV ctDNA is linked to tumor burden, disease stage, and metastasis.^34^ Previous studies have shown that detectible EoT and baseline HPV ctDNA values correlate well with tumor load and therefore HPV ctDNA dynamics can be used as a surrogate of treatment response.^18^ Here, by combining results from multiple data sets, we showed that measurement of residual disease following concurrent use of chemotherapy and radiation—chemoradiotherapy (CRT)—and neoadjuvant therapy accurately well predict survival in HPV‐associated cancers. Some evidence indicates that the cytotoxic effects of chemotherapy or radiation therapy seem to increase cell‐free DNA levels due to cellular senescence.^42^, ^43^ Moreover, other studies have indicated that levels of HPV ctDNA drop significantly in patients with (CC) and LACC during CRT.^32^, ^39^ This means that the sensitivity of HPV ctDNA detection at the end of CRT is not perfect in predicting recurrence. However, our pooled analysis demonstrated that measuring plasma/serum HPV ctDNA levels at the end of treatment is more effective in predicting recurrence compared to measuring levels at baseline. According to survival data, HPV ctDNA clearance after CRT and/or neoadjuvant therapy can outperform tumor response to treatment. Although further studies are needed, HPV ctDNA postulates as an early surrogate of recurrence prediction being a promising endpoint assessment in the neoadjuvant and CRT setting; those administrations are widely used for oropharynx, cervical, and anal canal cancers.^44^, ^45^, ^46^ Here, we showed that the predictive performance of HPV ctDNA for OPSCC was better than CC. It means that more focus is needed for HPV ctDNA in the decision‐making of postradiotherapy management of oropharyngeal cancer.

PET/CT is often used for long‐term follow‐up goals, treatment planning, and accurate prediction of treatment response in patients receiving radiation therapy (RT). Moreover, PET/CT is a routine tool for response evaluation after RT and follow‐up imaging for cancers with a high negative predictive value. Some unresolved issues, such as optimal uptaking of the probe, optimal cut‐off values (optimal threshold values that can be used to determine whether a tumor is responding to radiation therapy), and the optimal timing of PET/CT during RT in predicting the radiotherapy response of different tumors have diverse results and are still under research.^47^ RT response evaluation using PET/CT is currently undertaken 10 to 12 weeks post CRT and can be postponed to minimize false‐positive results; like in the presence of inflammations, as the most common cause of false‐positive PET/CT findings post‐chemotherapy and radiotherapy that need to be is resolved.^48^ Delaying PET/CT treatment response assessment would help decrease the frequency of false‐positive as well as false‐negative results. However, delaying PET‐CT treatment response assessment may lead to delayed salvage therapy and a poorer prognosis.^34^ However, PET/CT imaging is not merely a solution after radiotherapy and can be complemented with HPV ctDNA measurement to empower decision‐making in the radiation oncology field.^34^ We propose that positivity for HPV ctDNA at that end‐of‐treatment timepoint can outperform PET‐CT in predicting treatment failure after radiotherapy with or without chemotherapy in patients with HPV‐associated tumors, especially in HPV‐positive HNSCC tumors.

According to the risk ratio we found, patients with fast HPV ctDNA clearance and low recurrence risk after treatment could be considered for adaption of CRT with less‐intensive therapy in the last weeks of CRT, to avoid harmful side effects and unnecessary medications. Repeated time‐points may be needed to better predict relapse. As reported previously, two consecutively positive plasma HPV ctDNA detection in HNSCC patients during post‐treatment surveillance brought high (above 90%) positive and negative predictive values indicating its potential in facilitating the decision for salvage therapy.^35^ Although several patients have achieved complete clearance of HPV ctDNA only at the end of CRT and did not subsequently experience relapse, so positive HPV ctDNA detection during CRT did not appear to be a reliable predictor of relapse.^32^ This suggests that HPV ctDNA detection at the end of treatment timepoint has a superiority for earlier timepoints.

**Question:** If a tumor is predicted to have a good response to RT and/or CRT, it seems to be worth performing CRT or RT only as a treatment modality. Otherwise, if a tumor is predicted to be radio‐resistant, how we can predict the radiosensitivity of tumors using HPV ctDNA measurement remained to be evaluated.

### 4.1 Limitations

Since anal, penile, vaginal, and vulvar cancers are relatively rare, the potential role of HPV ctDNA in monitoring these cancers has yet to be fully evaluated. We were also unable to analyze cancer surveillance data according to tumor stage. No data were available regarding detectible HPV ctDNA at several time points, especially in mid‐treatment or weeks after treatment for real‐time ctDNA monitoring. Also, there were no qualitative attributes regarding HPV DNA fragments and HPV types in most of the studies and their implementation in clinical practice. Included studies had no relevant information regarding the immunohistochemical detection of p16INK4a (p16) as a reliable marker for active HPV infection.

## 5 CONCLUSIONS

In conclusion, ultrasensitive detection of HPV ctDNA can predict relapse or recurrence rate after treatment. Based on the obtained data, the end‐of‐treatment measurement of HPV ctDNA has superiority over baseline measurement in treatment monitoring and response assessment of HPV‐associated cancers. Also, OPSCC patients can ultimately benefit from HPV ctDNA measurement; so it is essential to incorporate HPV ctDNA measurement into the decision‐making of postradiotherapy management in OPSCC treatment settings to avoid unnecessary invasive procedures. Although the end‐of‐treatment timepoint sensitivity exceeded relapse prediction, however, a positive result might not guarantee subsequent recurrence prediction and could necessitate repeat testing or complemented with PET/CT scan. Future studies are needed for later time points measurements to guide real‐time treatment decision‐making of HPV‐associated cancers.

## AUTHOR CONTRIBUTIONS

**Abbas Karimi:** Conceptualization (equal); data curation (equal); investigation (equal); methodology (equal); project administration (equal); supervision (equal); writing – original draft (equal); writing – review and editing (equal). **Tohid JafariKoshki:** Data curation (equal); formal analysis (equal); investigation (equal); methodology (equal); validation (equal); writing – review and editing (equal). **Mojtaba Zehtabi:** Data curation (equal); investigation (equal); writing – review and editing (equal). **Farzaneh Kargar:** Investigation (equal). **Tarik Gheit:** Conceptualization (equal); investigation (equal); supervision (equal); writing – review and editing (equal).

## CONFLICT OF INTEREST STATEMENT

The authors declare that there are no conflicts of interest.

## DISCLAIMER

Where authors are identified as personnel of the International Agency for Research on Cancer/World Health Organization, the authors alone are responsible for the views expressed in this article and they do not necessarily represent the decisions, policy, or views of the International Agency for Research on Cancer/World Health Organization.

## Data Availability

The data that support the findings of this study are available from corresponding authors but restrictions apply to the availability of these data, which were used under license for the current study, and so are not publicly available. However, data are available from the authors upon reasonable request and with permission of corresponding authors.
